# Supplementary material for: Programmable integrated photonics for topological Hamiltonians
Source: Nat Commun. 2024 Jan 20;15:629. doi: 10.1038/s41467-024-44939-3 (PMC10799881; doi:10.1038/s41467-024-44939-3)
Supplement: Supplementary file 1 — Supplementary Information [file 41467_2024_44939_MOESM1_ESM.pdf]

Supplementary Information for "Programmable Integrated Photonics for Topological Hamiltonians"

Mehmet Berkay On[1,2], Farshid Ashtiani[1], David Sanchez-Jacome[3], Daniel Perez-Lopez[3], S. J. Ben Yoo[2], and Andrea Blanco-Redondo[1,4,*]

[1]*Nokia Bell Labs, 600 Mountain Ave, New Providence, NJ 07974, USA*
[2]*University of California Davis, Department of Electrical and Computer Engineering, One Shields Avenue, Davis, CA 95616, USA*
[3]*iPronics Programmable Photonics, Avenida Blasco Ibanez 25, Valencia 46010, Spain*
[4]*CREOL, The College of Optics and Photonics, University of Central Florida, Orlando, FL 32816, USA*
*andrea.blancoredondo@ucf.edu

Supplementary Note 1: Experimental Setup Details and Programmable Mesh Calibration

All the hexagonal resonators programmed in the mesh consist of six ideally equal PUCs and should, therefore, have equal resonant frequencies. However, fabrication variations on the silicon waveguide cause phase errors [1]. Additionally, local temperature variations on the processor chip shift the resonance wavelength of the individual resonators [2]. The processor is calibrated once as part of its first validation process. The calibration extracts the passive offset of each programmable unit cell that is generated due to design and fabrication errors. This data is stored in the logic unit of the processor and remains fixed in time and for a considerable $\pm 5°C$ chip base temperature range therefore there's no need for further calibration of the device after the first routine. Even though the first validation process compensates phase offset for each PUC individually, we observed that resonance wavelengths of the resonators vary, as shown in Fig.S2a because small fabrication imperfections and temperature differences lead to misalignment between the resonances of each ring which consists of six PUCs. Specifically, despite the temperature of the processor being controlled via a thermo-electric cooler (TEC), the chip can undergo temperature drifts of $\pm 0.5°C$ due to large temperature room fluctuations of $\pm 6°C$ (e.g. air conditioning on and off). Such temperature drifts could translate in ± 5 pm shifts in the resonant frequency of a ring. By using two thermoptical phase shifters in the 2×2 programmable unit cell (PUC), (equation (1-3) in the main text), we can tune the resonance wavelength of the individual resonators without disturbing power coupling ratios. We run the calibration procedure once before the 1D SSH model measurements and do not recalibrate during the measurements. First, we configure the programmable mesh so that the resonators can be excited and monitored one by one. Then, one of the dedicated PUC in each hexagonal resonator is tuned adaptively to match the resonance wavelength of every hexagonal resonator as shown in Fig.S2b.

Fig.S1 illustrated the experimental setup, excitation port, and monitoring ports. Because the output ports are located only on the edges of the mesh, the power tapped out of certain resonators (specifically resonators 3, 5, and 7) need to travel through multiple external PUCs to be monitored. Since PUCs have 0.5 ± 0.05 dB insertion loss in this specific hardware implementation, longer paths to the photodiodes result in non-negligible additional loss. Therefore, we recorded the monitored power at the resonance wavelength for each resonator and subsequently postprocessed the measurements of the rings in the 1D SSH model to compensate for the additional extrinsic loss associated with the longer paths to reach PD-3, PD-5, and PD-7.

We use a C-band tunable laser (TL) with 0.003 nm tuning resolution. The TL sweep from 1549.9 nm to 1550.2 nm and monitoring photodetectors (PD) measure the optical power values. The sensitivity of the PDs is -70 dbm. For monitoring purposes, we tapped 1% of the power by one of the available PUC of the hexagonal resonator. SmartLight Processor is programmed through the Python interface developed

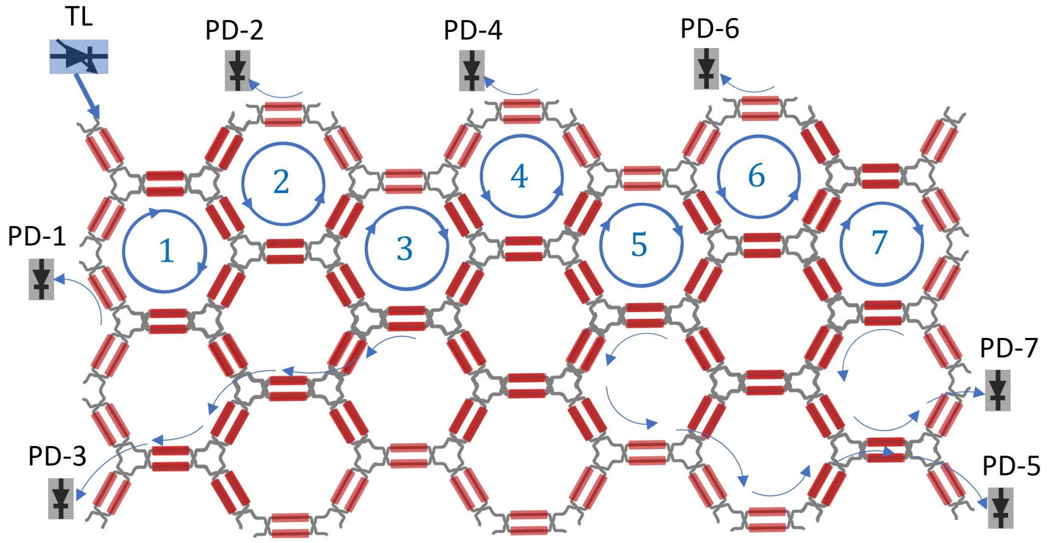

Fig. S1: Schematic of the programmable mesh on iPronics's SmartLight Processor and reconfiguration for 7 coupled ring resonators, TL: off-chip tunable laser, PD: off-chip photodetector.

by iPronics. Through the interface power coupling ratio ($\sin^2(\Delta)$) of the PUCs and cross-phase values (θ) are set. The power coupling ratio is a value between "0" and "1". "0" represents the bar state for the 2×2 unit, while "1" is the cross state. The coupling ratios between the resonators are set accordingly. We refer to the following formula derived similarly in the supplemental document of the article [3],

$$k = \sin^2(\Delta)\frac{FSR}{4} \tag{1}$$

to calculate coupling rates presented in the main text. We measured the free-spectral range (FSR) of the hexagonal resonators as 14.6 GHz, spacing between consecutive resonances. We set input PUC's power coupling ratio as 0.8 ($k_{in} = 2.9GHz$) to inject most of the TL output power into the 1D SSH model.

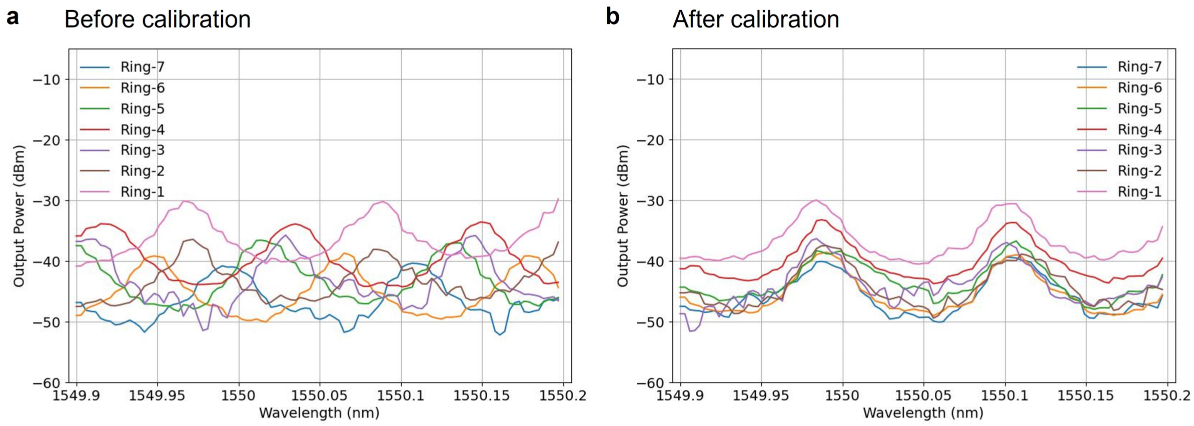

Fig. S2: Individually monitored ring resonators **a** before and **b** after the resonance wavelength calibration.

Supplementary Note 2: Comparison between Simulated and Measured 1D SSH Model

We simulated the coupled ring resonators and compared simulation results with measurements of the 1D SSH model from the programmable hardware. The simulation parameters, insertion loss per PUC, laser wavelength, coupling, and monitoring PUC locations are set accordingly to match the hardware

implementation. Fig.S3a, b presents power distribution at the resonance wavelength from the simulations and measurements. We fitted the function $f(x) = c_1 e^{-\alpha x} + c_2$ on the power values of the odd rings. Here, x is the odd rings' indices. The parameter α refers to the penetration depth of the edge states.

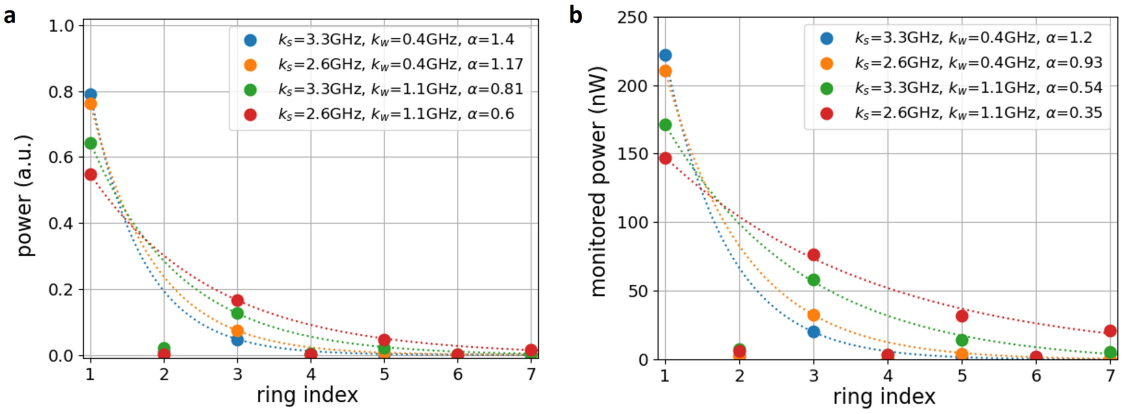

Fig. S3: Powers at coupled ring resonators from **a** simulator and **b** measured on hardware. Dashed traces are fitting function $f(x) = c_1 e^{-\alpha x} + c_2$ on the odd rings of 1D SSH model.

Supplementary Note 3: Simulation Details and Path-Symmetric 2D Kagome Lattice

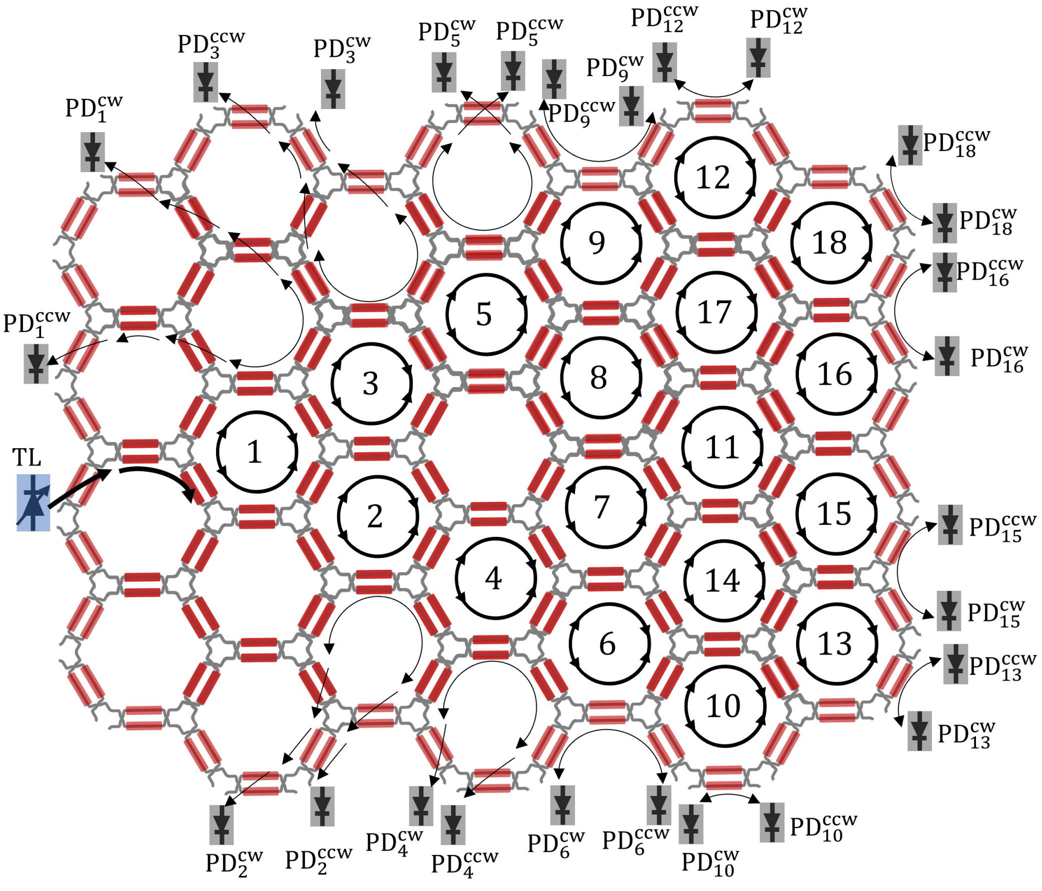

Fig. S4: Schematic of the simulated path-symmetric 2D Kagome lattice on the programmable mesh.

We observed that various implementations of the 2D breathing Kagome lattice might result in slightly

different power localization even though all the simulation settings are equal. Optical path differences between the input PUC and the corner resonators are the main cause of these variations. To verify this, we implemented the symmetric configuration in Fig.S4, which resembles more closely the ideal 2D Kagome lattice in terms of light paths and results in a more equal power distribution on the corner rings. Note that at the time of these studies, the simulator can only achieve a maximum of 5 hexagons in the vertical direction. Therefore, the power on ring-17 and ring-14 in this symmetric configuration could not be monitored, which is the reason for showing the more asymmetric case in the main text. Recent work by Sanchez *et. al* [4] opens a path for arbitrary size programmable mesh simulations in the future.

Only the 2D Kagome lattice resonators' PUCs in the simulator have 0.1 dB insertion loss, while the routing PUCs for monitoring paths are lossless. The tuning precision of the laser is 0.2pm ($\sim 25MHz$) around 1550 nm. The FSR of the simulated resonators is 12.5 GHz. The coupling rate for the input PUC to the system is set to $k_{in} = 1.6$ GHz. Fig.S5 presents the simulation results for the path-symmetric lattice. While we observed similar behavior of "degeneracy" in the spectrum, corner rings 13 and 18 have more localized power than the main text Fig.6.

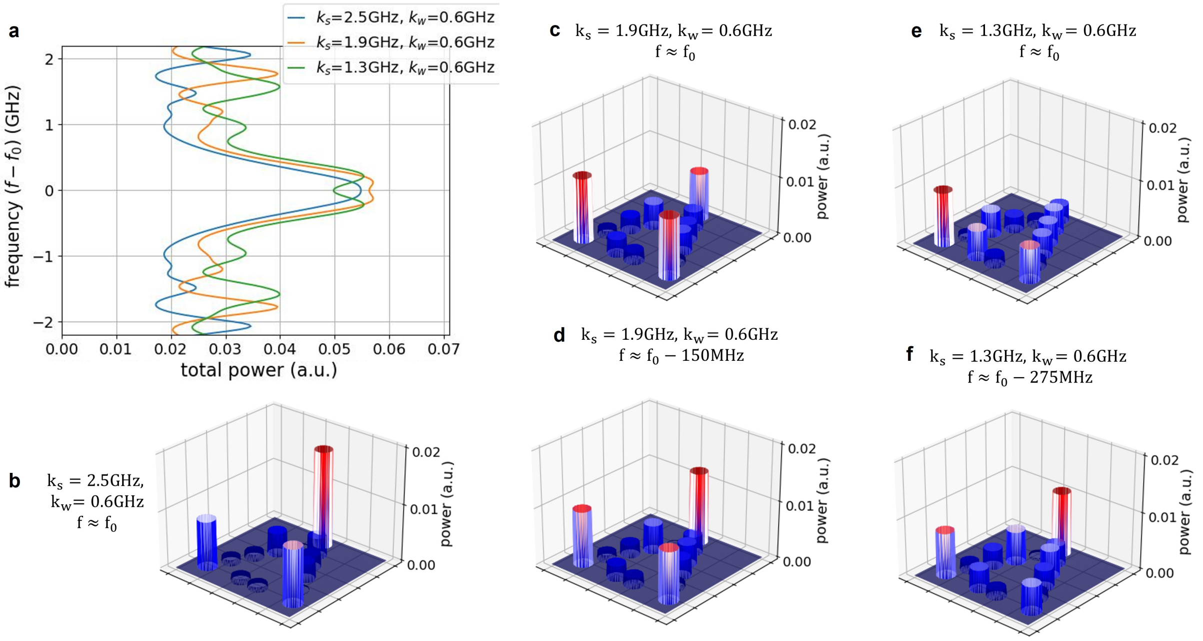

Fig. S5: **a** Simulated spectrum with various dimerizations, **b-f** Simulated power distribution on the Kagome lattice wit various dimerizations and frequencies.

References

[1] Goh, T., Suzuki, S. & Sugita, A. Estimation of waveguide phase error in silica-based waveguides. *Journal of Lightwave Technology* **15**, 2107–2113 (1997).

[2] Bogaerts, W., Fiers, M. & Dumon, P. Design challenges in silicon photonics. *IEEE Journal of Selected Topics in Quantum Electronics* **20**, 1–8 (2014).

[3] Hafezi, M., Mittal, S., Fan, J., Migdall, A. & Taylor, J. M. Imaging topological edge states in silicon photonics. *Nature Photonics 2013 7:12* **7**, 1001–1005 (2013).

[4] Sánchez, E., López, A. & Pérez-López, D. Simulation of Highly Coupled Programmable Photonic Circuits. *Journal of Lightwave Technology, Vol. 40, Issue 19, pp. 6423-6434* **40**, 6423–6434 (2022).
